# Sex-specific glomerular filtration rate changes in response to acute hemoglobin exposure

**DOI:** 10.1016/j.biopha.2025.118461

**Published:** 2025-08-15

**Authors:** Daniela Lucas, Cynthia R. Muller, Carlos Munoz, Quintin O’Boyle, Andre F. Palmer, Pedro Cabrales

**Affiliations:** a Shu Chien-Gene Lay Department of Bioengineering - University of California, San Diego, CA, United States; b William G. Lowrie Department of Chemical and Biomolecular Engineering, The Ohio State University, Columbus, OH, United States

**Keywords:** Hemoglobin toxicity, Acute kidney injury (AKI), Sex differences, Estrous cycle, Oxidative stress, Glomerular filtration rate, Estrogen

## Abstract

Hemoglobin (Hb) toxicity is a known contributor to acute kidney injury (AKI), particularly under hemolytic conditions where cell-free Hb is present in circulation. When the endogenous Hb scavenger protein haptoglobin becomes saturated with cognate ligand Hb, excess unbound cell-free Hb extravasates into tissues, including the kidneys, leading to oxidative stress, inflammation, and impaired renal function. Although sex-based differences in AKI susceptibility have been observed, the protective role of hormones in modulating the severity of Hb-induced kidney injury remains unclear. In this study, we evaluated the impact of biological sex, specifically estrous cycle status, on renal responses to acute Hb exposure. Glomerular filtration rate (GFR) was measured noninvasively using the Medibeacon transdermal device that detects clearance of a fluorescent tracer (FITC-Sinistrin). Across all groups, the GFR declined at 2 h post-exposure to Hb; however, only females in estrous fully recovered by 24 h. Males and non-estrous females showed sustained reductions in GFR, suggesting impaired renal recovery. Injury biomarkers for kidney, liver, urine, and heart, including KIM-1, bilirubin, creatinine, troponin, and others, showed significant improvement in females during estrous compared to males and non-estrous females. All groups exhibited some degree of toxicity from the Hb, as demonstrated by elevated levels of markers compared to the control group. Nonetheless, these findings suggest that the hormonal levels related to the estrous cycle protect against cell-free Hb-induced acute renal and cardiac injury, potentially through modulation of inflammatory signaling pathways. Understanding sex- and hormone-dependent responses to Hb toxicity is critical for developing targeted therapies.

## 1. Introduction

Hemoglobin (Hb) toxicity is a known contributor to kidney injury, particularly in hemolytic conditions where cell-free Hb is constantly released into the circulation [1,2]. These conditions can result from diseases such as hemolytic anemias or therapeutic interventions, including extracorporeal blood circulation devices or certain chemotherapy drugs [3–6]. Once outside the RBC, Hb tetramers (α_2_β_2_, MW ~ 64 kDa) are unstable and rapidly dissociate into αβ dimers (MW ~ 32 kDa) and even α and β monomers (MW ~ 16 kDa). These subunits are small enough to cross endothelial barriers and accumulate in the perivascular space and vital organs [1,7]. Outside the protective environment of the RBC, Hb is prone to oxidative modifications, during which the heme iron is oxidized from its ferrous (Fe^2+^) to ferric (Fe^3+^) state, forming methemoglobin (MetHb). MetHb has a reduced affinity for globin chains, increasing the likelihood of heme dissociation and release [8]. Free heme is highly lipophilic and catalytically active, which can generate reactive oxygen species (ROS), and activate pro-inflammatory signaling pathways [9,10]. This degradation cascade is a key contributor to Hb-induced tissue injury and is especially detrimental to the cardiovascular system and organs with high perfusion and filtration activity, such as the kidney. [7,11]

The kidneys’ fenestrated capillaries, specifically in the glomeruli, play a crucial role in the filtration of water and small solutes while preventing the larger proteins from crossing into the Bowman’s capsule. Under normal conditions, protective plasma proteins like haptoglobin and hemopexin scavenge free Hb and heme to maintain homeostasis [12,13]. However, when these systems are overwhelmed, free Hb and heme accumulate and trigger oxidative stress, endothelial dysfunction, and tubular injury [1,14–17]. Moreover, these effects are known to activate innate immune pathways, including the Nod-like Receptors such as the NLRP3 inflammasome [18–21]. This multiprotein complex cascades towards cell death activation due to inflammation and is partially responsible for cell death in kidney injury following various toxic and ischemic insults [22,23]. A critical question remains: at what threshold of kidney damage does Hb-induced toxicity compromise the glomerular filtration rate (GFR) and lead to long-term renal complications? Although a correlation between acute kidney injury (AKI) and cell-free plasma Hb has been established, research on the direct effects of Hb on GFR is limited [6,24]. Moreover, the influence of biological sex and hormonal status on the susceptibility to Hb-mediated renal damage is not fully understood.

Emerging evidence suggests that biological sex is a key determinant of kidney injury outcomes and may influence susceptibility to Hb toxicity. Females tend to experience less frequent and less severe AKIs with reduced inflammatory responses compared to males [25–27], a difference that has been partially attributed to sex chromosomes and hormone-mediated protection [28–30]. For example, X-linked genes may enhance cellular resilience through effects on nephron transporter activity and damage-response signaling pathways [30]. More prominently, estrogen has been shown to attenuate renal injury by modulating oxidative stress and suppressing inflammatory cascades, including those involving the inflammasome [28,29]. Conversely, testosterone has been implicated in exacerbating renal injury across several disease models [31–34]. These findings suggest that sex hormones could influence how the kidney responds to Hb-induced injury, though this remains understudied in the context of acute hemolysis.

In this study, we investigated the acute effects of acute cell-free Hb exposure on renal function and molecular markers of kidney injury in male, estrous-phase female, and non-estrous-phase female animal subjects. We assessed GFR to evaluate functional impairment and examined molecular signatures of kidney damage to determine differential responses based on biological sex and hormonal status. To evaluate kidney injury at the molecular level, we selected kidney injury molecule-1 (KIM-1) and neutrophil gelatinase-associated lipocalin (NGAL) as biomarkers, among others. These markers are highly sensitive indicators of early proximal tubular damage and have been validated extensively in models of nephrotoxicity, including hemolysis and heme-induced renal injury [35–37]. KIM-1 is a transmembrane protein upregulated in injured tubular epithelial cells, while NGAL is released rapidly in response to tubular stress and oxidative injury [38,39]. Their early expression precedes histopathological changes, allowing sensitive detection of acute kidney injury relevant to Hb toxicity. By identifying potential sex-specific susceptibilities to cell free Hb-induced AKI, our findings aim to inform future precision medicine approaches for mitigating renal complications associated with hemolytic disorders.

## 2. Methods

### 2.1. Hb Purification

Expired human red blood cell units were acquired from the Transfusion Services at The Ohio State University’s Wexner Medical Center. The acellular free Hb used in these studies was obtained through purifying human RBCs via tangential flow filtration, following a previously established method [40]. Hb concentration was measured using spectrophotometric analysis [41].

### 2.2. Animal Preparation

Experiments were conducted using male and female Sprague-Dawley rats (Harlan Laboratories, Indianapolis, IN) with weights ranging from 150 to 200 g. Animal care and handling adhered to the NIH Guide for the Care and Use of Laboratory Animals, and the experimental protocol was approved by the UCSD Institutional Animal Care and Use Committee. Animals were anesthetized with isoflurane (5 %/vol for induction, 2.5 %/vol for maintenance, Drägerwerk AG, Lübeck, Germany) and underwent left femoral artery catheterization. The animals were positioned supine on a heating pad to maintain their core body temperature at 37°C and were allowed to breathe freely from a nosecone. After surgery, the isoflurane concentration was reduced to 1.5 %/vol. Anesthesia depth was continually monitored using a toe pinch, and the isoflurane concentration was adjusted if necessary.

### 2.3. Estrous Cycle Determination

Female Sprague Dawley rats underwent vaginal lavage at the start of experimentation to assess the phase of the estrous cycle. While holding the rat securely by the base of the tail to expose the vaginal opening, a pipette tip containing 50 μL of sterile saline solution was gently inserted into the vaginal canal. The vaginal lumen was rinsed by introducing the saline with repeated aspiration and agitation using the pipette tip. The collected lavage fluid was then placed on a microscope slide, mixed with an equal volume of crystal violet solution (Polysciences, Cat# 0022), and covered with a coverslip. Samples were examined under a light microscope for cytological evaluation.

Estrous phase was determined based on standard cytological criteria, including the relative presence of nucleated epithelial cells, cornified epithelial cells, and leukocytes. Proestrus was identified by predominantly nucleated epithelial cells, estrus by mostly cornified epithelial cells, metestrus by a mix of cornified cells and leukocytes, and diestrus by abundant leukocytes. Classification was conducted immediately before Hb administration to match hormonal status to the acute response window. Given the short half-life of cell-free Hb and the transient nature of estrogen-mediated protection, this time point was validate via a second smear collected at 24 h post-Hb administration. The second smear were used to also confirm that each animal’s estrous cycle progressed in a physiological sequence.

### 2.4. Blood Exchange Transfusion Protocol

Anesthetized rats underwent a 20 % exchange transfusion of their estimated blood volume with ultrapure Hb solution. The exchange volume was calculated as 7.0 % of the animal’s body weight. The transfusion was performed manually by alternating blood withdrawal and acellular Hb solution infusion (5 g/dL). Briefly, using the catheter, 0.5 mL of blood was withdrawn over approximately 5 min, followed by the infusion of 0.5 mL of the Hb solution at the same rate. A small volume of saline was then injected to ensure complete delivery of the solution and to rinse the catheter. This cycle was repeated until the calculated exchange transfusion volume was achieved.

### 2.5. Kidney Function

Anesthetized rats were placed in a prone position on a heated pad for placement of a transdermal GFR monitoring device (MediBeacon, St. Louis, MO, USA). The skin was closely shaved using a razor blade and cleaned with 70 % ethanol. The transdermal GFR monitoring device was placed on one side of the adhesive patch, ensuring that the LEDs were positioned directly over the clear window. The battery was connected to the device, and data acquisition started once the red LED began blinking. The device was securely attached to the shaved skin, with the LED window positioned over the abdomen. The blinking LED turned green upon successful skin detection. To minimize light interference, the lower part of the animal’s body was covered with aluminum foil. The device was left in place for 5 min before FITC-sinistrin (MediBeacon) injection to establish a baseline signal. Following the 5-minute stabilization period, and after the device ceased blinking red, FITC-sinistrin was prepared in a 1 mL syringe at a dose of 70 mg/kg (diluted 70 mg of FITC-Sinistrin Medibeacon in 1 mL of sterile saline), rounded to the nearest 10 μL. The solution was administered via the femoral catheter placed during surgery. After injection, the catheter was flushed with saline to ensure complete circulation of the FITC-sinistrin. GFR measurements were recorded over a 2-hour period, after which the device was removed. At the end of the measurement period, the femoral artery was ligated using a 6–0 silk suture, and the animal was transferred to a heated pad for recovery from anesthesia. GFR measurements were repeated 24 h later via catheterization of the right femoral artery.

Timepoints of 2 h and 24 h post-Hb exposure were selected to capture key phases of the Hb circulation time and the acute renal response to acellular Hb. The 2-hour timepoint represents the early injury peak, coinciding with elevated levels of circulating cell-free Hb. The 24-hour timepoint reflects the early recovery phase, when circulating Hb is largely cleared, and renal function begin to normalize. These time points were chosen based on the known short half-life of cell-free Hb and its transient renal toxicity.

### 2.6. Systemic Hemodynamics Parameters

Mean arterial pressure (MAP) and heart rate (HR) were recorded continuously from the femoral artery using a PowerLab 8/30 data acquisition system and analyzed with LabChart 7 software (ADInstruments, Colorado Springs, CO). Arterial blood was collected in heparinized glass capillary tubes (65 μL) and immediately analyzed for blood chemistry and gases (ABL90; Radiometer America, Brea, CA). To confirm circulating Hb concentrations following infusion, whole blood was collected via the femoral artery into heparinized capillary tubes at baseline, 2 h, 4 h, and 24 h post-infusion. Samples were centrifuged to separate plasma and cells, and then the plasma Hb concentrations were measured using a HemoCue^®^ Plasma/Low Hb photometer (HemoCue AB, Ängelholm, Sweden).

### 2.7. Markers of Organ Damage and Inflammation

After the experimental protocol, the organs were harvested for molecular marker and histological analysis. Kidney injury molecule-1 (KIM-1) was measured in kidney tissue homogenates using an ELISA kit (ERHAVCR1, Thermo Fisher, Waltham, MA). Kidney and plasma cytokine levels of interleukin-6 (IL-6) and interleukin-10 (IL-10) were quantified using ELISA kits (BMS625 and BMS629, respectively, Thermo Fisher). Interleukin-1 beta (IL-1β) and interleukin-18 (IL-18) were measured using ELISA kits (BMS6002 and KRC2341, Thermo Fisher). Urinary creatinine was measured using kit EIACUN (Thermo Fisher). Neutrophil gelatinase-associated lipocalin (uNGAL) and albumin were measured using kits ERLCN2 and EEL124, respectively (Thermo Fisher). Plasma cardiac troponin I was quantified using an ELISA kit (EEL112, Thermo Fisher). Liver function was measured from plasma bilirubin using kit NBP2-69939 (Novus Biologicals), and aspartate transaminase (AST) using kit EEL086 (Thermo Fisher). Blood urea nitrogen (BUN) was measured using kit 0544-MBS2611086-96 (Biomeda Corp). Heme oxygenase-1 (HO-1) was quantified using kit EEL141 (Thermo Fisher). Urinary iron was assessed using kit ab83366, and plasma heme was measured using kit ab272534 (Abcam).

### 2.8. Statistical Analysis

Each experimental group consisted of 7 animals. Data are presented as the mean ± standard deviation. Hemodynamic parameters and GFR were analyzed using a two-way ANOVA to compare groups and experimental time points, with *post hoc* comparisons performed using Tukey’s HSD test to determine significant differences. For organ inflammatory and injury markers, a one-way ANOVA was used to compare group means, followed by Tukey’s HSD test for post hoc analysis. A P-value of < 0.05 was considered statistically significant. All analyses were conducted using GraphPad Prism 9 (GraphPad Software, Inc., San Diego, CA)

## 3. Results

### 3.1. Mean Arterial Pressure and Heart Rate

**Fig. 1.**
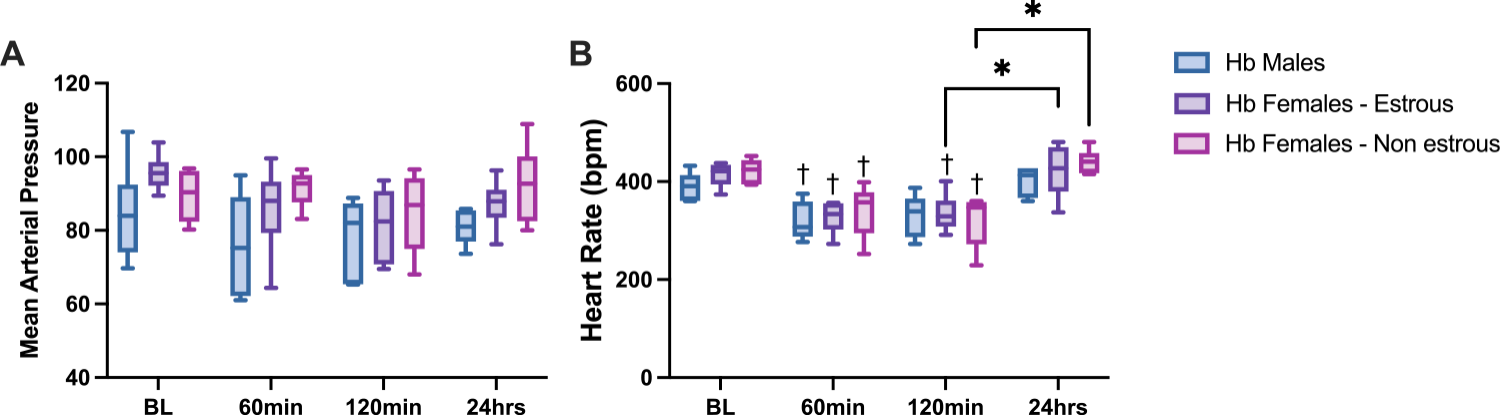
Hemodynamic Measurements: Mean Arterial Pressure and Heart Rate. Mean arterial pressure and heart rate showed no significant difference between groups at baseline or after the 20 % blood exchange with hemoglobin (Hb). However, Hb exposure decreases heart rate 60 mins and 120 mins after exchange transfusion. **A)** Mean arterial pressure for the duration of the experimental protocol shows no significance between groups **B)** Heart rate for the duration of the experimental protocol shows no significance between groups, but all groups show a reduction in heart rate due to Hb infusion. Data are presented as boxplots, with median and whiskers representing the data range. Statistical significance (p < 0.05) between experimental groups is marked by * and compared to the control group is indicated by †.

MAP and HR results are presented in Fig. 1, showing measurements taken at 20-minute intervals from the start of the experiment, beginning with initial anesthesia and continuing for two hours. Additional measurements were recorded at 24 h, following re-anesthesia for the final GFR assessment. The data indicate no significant differences in MAP and HR between groups. MAP remained stable through all time points and across all groups, showing no significant difference. Although Hb is known to increase MAP, this effect is likely masked by systemic anesthesia. However, a significant drop in HR was observed during the experiment compared to baseline, which is expected due to Hb exposure. This decrease in HR was temporary, as animals returned to baseline values after the 24-hour recovery time point.

### 3.2. Plasma Hemoglobin Concentrations

To confirm circulating acellular Hb across groups, plasma Hb concentrations were measured following exchange transfusion (see Supplementary Figure S1). No significant differences were observed between groups, confirming that all animals received comparable amounts of cell-free Hb levels.

### 3.3. Glomerular Filtration Rate

**Fig. 2.**
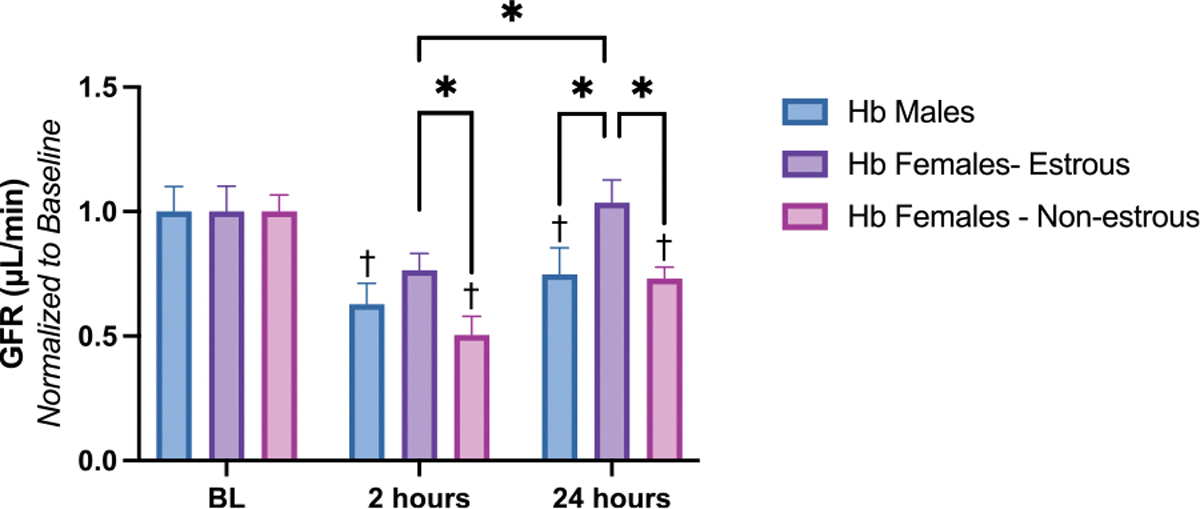
Glomerular Filtration Rate (GFR). GFR is represented for 2 timepoints in the experimental protocol, 2 and 24 h after Hb exchange transfusion and compared to the baseline values for each group. The females in estrous cycle exhibited the least significant impairment of GFR at 2 h with a full recovery to BL at 24 h compared to males and females in non-estrous cycle. Data are presented as column bar plots, with standard deviation representing the data range. Statistical significance (p < 0.05) between experimental groups is marked by * and compared to the control group is indicated by †.

The GFR results are presented in Fig. 2. Fig. 2 shows GFR measurements at 2 h and 24 h post-exchange transfusion, normalized to each animal’s baseline value. Baseline GFR values were not statistically significantly different between males, estrous-phase females, and non-estrous-phase females (see Supplemental Figure S1). At the 2-hour mark, all animals exhibited a decline in GFR. However, females in the estrous cycle did not experience a significant drop compared to their control group, whereas males and non-estrous females showed a significant decrease. By 24 h, GFR fully recovered in estrous females, while it remained significantly lower in males and non-estrous females. These two groups exhibited a significant impairment in recovery compared to estrous females and their respective controls.

It is important to note that estrogen levels were measured in both estrous and non-estrous females. Estrous females exhibited higher estrogen concentrations (0.07 ± 0.02 ng/mL) compared to non-estrous females (0.03 ± 0.00 ng/mL), highlighting the influence of estrogen hormone levels on the observed outcomes. The lack of variation in the non-estrous group reflects values near or below the assay’s detection threshold.

### 3.4. Organ Inflammation and Injury Biomarkers

The tissues of interest, including kidney, urine, and plasma, were harvested and subjected to analysis of inflammatory and injury biomarkers via ELISAs.

**Fig. 3.**
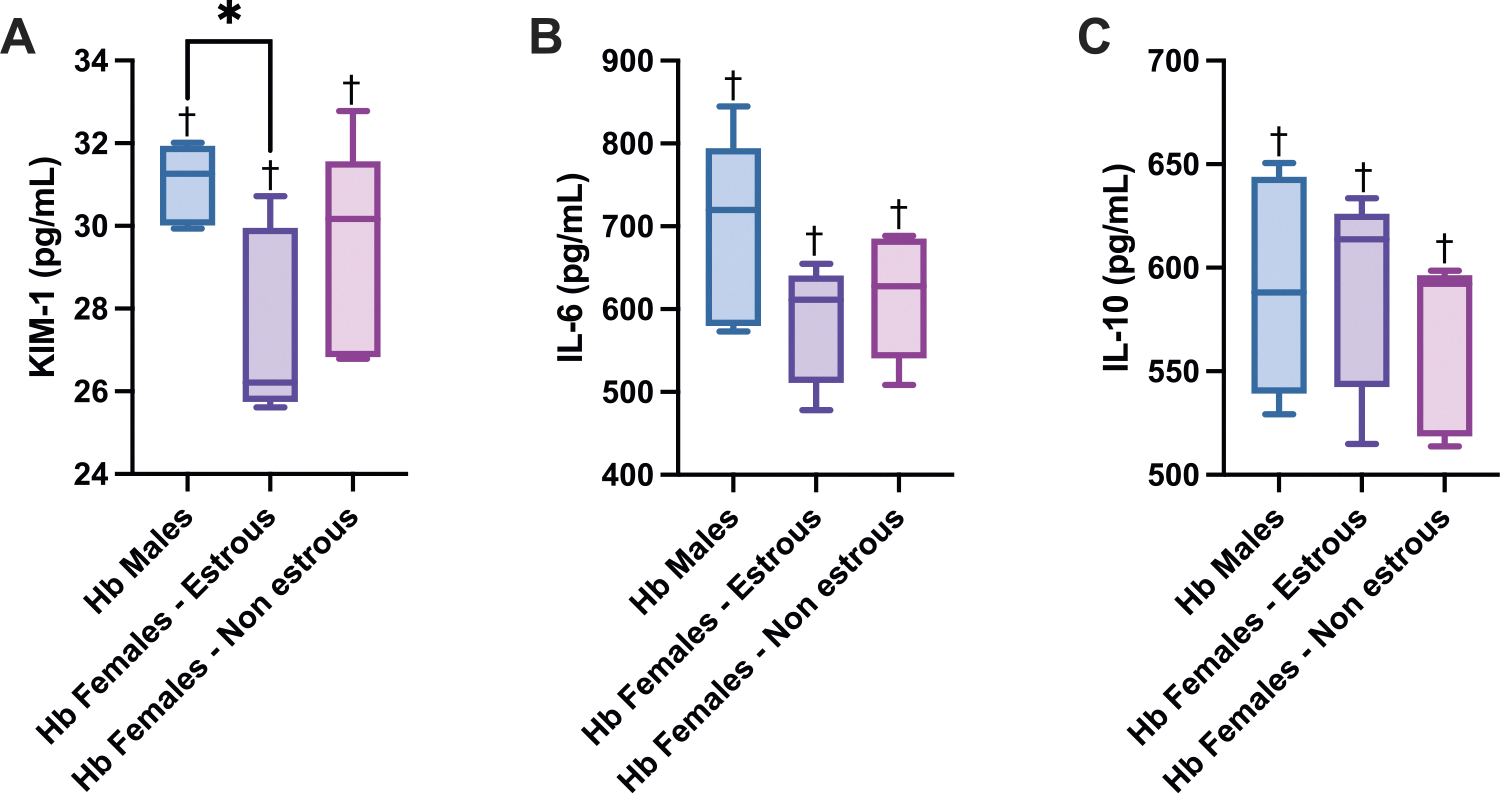
Kidney Injury and Inflammation Biomarkers. Renal injury marker levels of: (A) kidney injury marker KIM-1, (B) pro-inflammatory cytokine IL-6, and (C) anti-inflammatory cytokine IL-10 24 h after Hb infusion in male, estrous female, and non-estrous female rats. Hb-exposed males showed significantly elevated KIM-1 levels compared to estrous females. All groups displayed significant increases in KIM-1, IL-6, and IL-10 relative to their respective controls. Statistical significance (p < 0.05) between experimental groups is marked by * and compared to the control group is indicated by †.

To evaluate kidney injury and inflammation following Hb exposure, circulating levels of KIM-1, IL-6, and IL-10 were assessed (Fig. 3). Hb-exposed males exhibited significantly higher KIM-1 levels compared to estrous females, indicating greater renal injury. While IL-6 and IL-10 levels did not differ significantly between sex and estrous subgroups, all groups demonstrated significant elevations in these markers relative to their respective controls, suggesting a robust inflammatory response to acellular Hb.

**Fig. 4.**
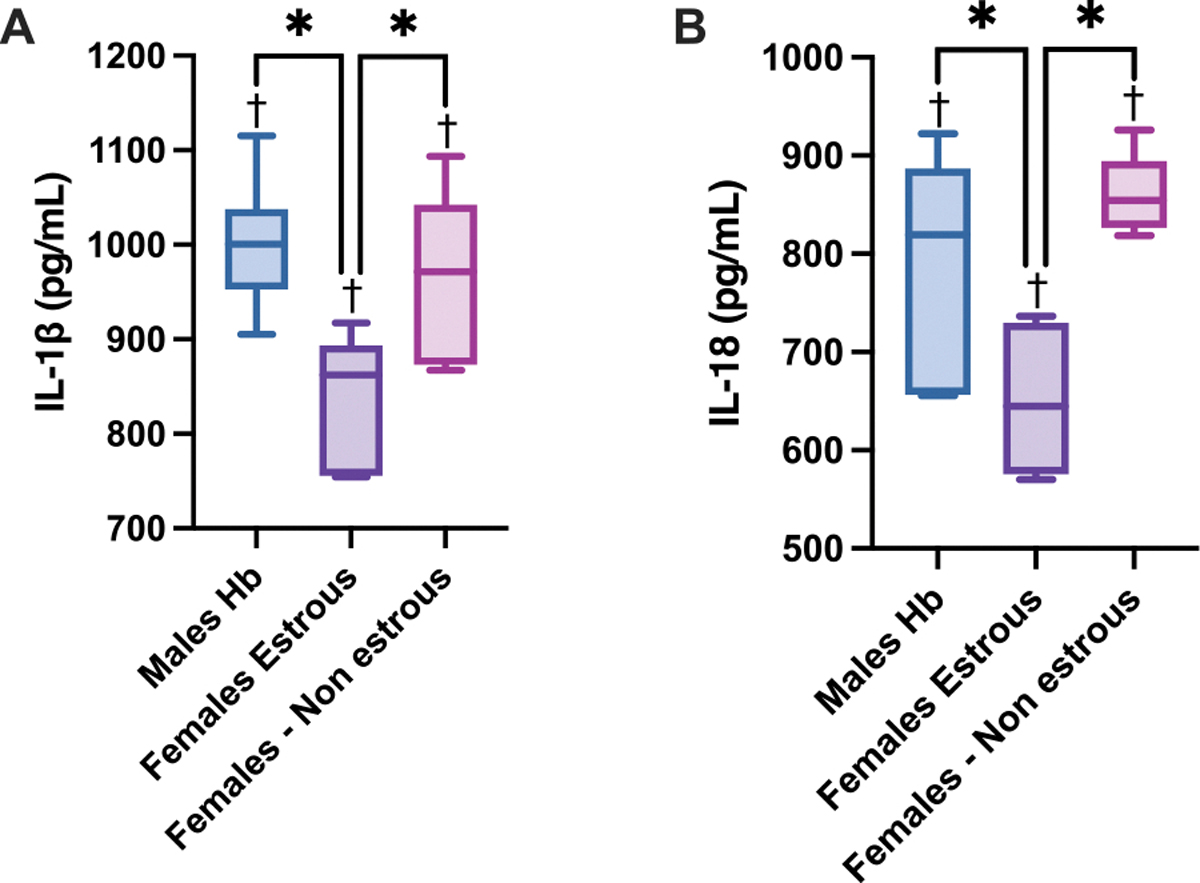
Quantification of Cleaved Interleukin Inflammatory Signals in NLRP3 Pathway. Inflammasome pathway interleukin signaling for A) IL-1β and B) IL-18 levels (pg/mL) 24 h after Hb infusion in male, estrous female, and non-estrous female rats. IL-1β levels and IL-18 levels were significantly reduced in estrous females relative to both males and non-estrous females, suggesting reduced activation of the NLRP3 pathway during the estrous cycle. Statistical significance (p < 0.05) between experimental groups is marked by * and compared to the control group is indicated by †.

To evaluate NLPR3 pathway activation, IL-1β and IL-18 levels were measured (Fig. 4). IL-1β levels were significantly lower in estrous females compared to both males and non-estrous females. Similarly, IL-18 levels were significantly decreased in estrous females compared to males and non-estrous females. Both IL-1β and IL-18 are key pro-inflammatory cytokines activated through the NLRP3 inflammasome pathway, which plays a critical role in mediating inflammation and promoting cell death. All markers showed significant differences compared to each group’s respective control. The observed reduction in these cytokines in estrous females suggests that the estrous cycle may confer protection against Hb-induced inflammasome activation, potentially mitigating renal injury and cell death.

**Fig. 5.**
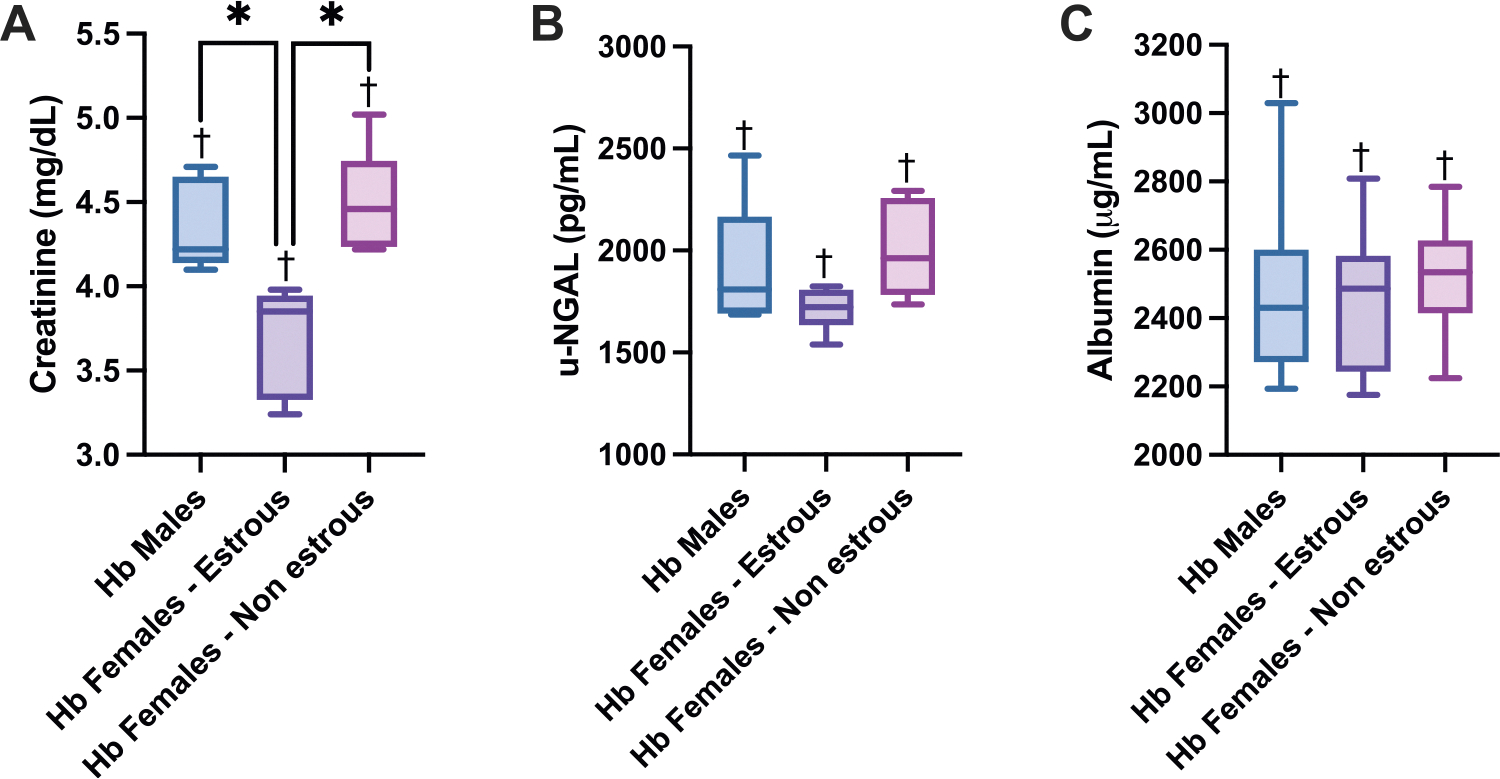
Urine Injury and Inflammation Biomarkers. Urine injury markers 24 h after Hb infusion in male, estrous female, and non-estrous female rats. The figures depict A) urine creatinine (mg/dL), B) urinary NGAL (ng/mL), and C) albumin (ng/mL) levels in males, females in estrous, and females in non-estrous stages. Plasma creatinine was significantly elevated in males and non-estrous females compared to estrous females and showed a more modest increase in estrous females, indicating partial protection from Hb exposure. Urinary NGAL levels showed no significant differences across groups, though trends mirrored those observed in creatinine levels. Statistical significance (p < 0.05) between experimental groups is marked by * and compared to the control group is indicated by †.

To evaluate kidney function via urine excretion, creatinine, uNGAL, and albumin were measured (Fig. 5). Females in the estrous phase exhibited lower levels of urine injury and inflammation markers compared to males and non-estrous females. Urine creatinine levels were significantly lower in estrous females compared to both males and non-estrous females, suggesting improved kidney function. While not statistically significant, uNGAL levels followed a similar trend, with estrous females exhibiting lower levels than the other groups. However, albumin content in the urine remained constant amongst groups, having no significance against each other. All markers showed significant differences compared to their respective controls within each group.

To further explore the relationship between functional impairment and molecular injury, we performed correlation analyses between GFR and selected kidney injury markers across all experimental groups (see Supplementary Figure S3; All correlations were statistically significant, p < 0.05). Scatter plots revealed a strong negative correlation between GFR and KIM-1, IL-18, and urinary creatinine. These results indicate that as GFR declined, levels of these biomarkers increased, supporting the link between functional loss and inflammatory tubular injury.

**Fig. 6.**
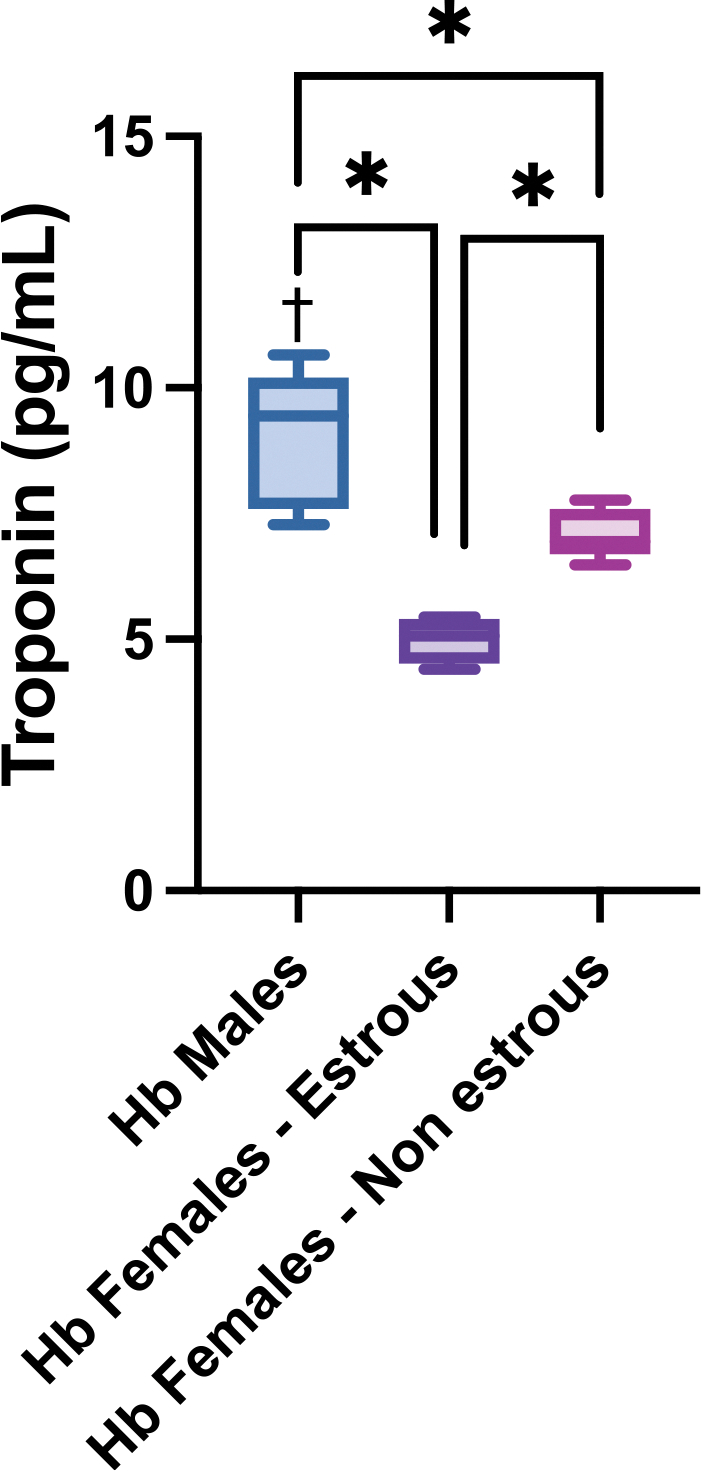
Plasma Troponin Levels. Circulating cardiac troponin levels 24 h after Hb infusion in male, estrous female, and non-estrous female rats. Circulating troponin was significantly elevated in Hb-exposed males compared to both estrous and non-estrous females. Non-estrous and estrous female groups also showed a significant difference from each other. Statistical significance (p < 0.05) between experimental groups is marked by * and compared to the control group is indicated by †.

To assess the multi-organ toxicity aspect of Hb, cardiac injury was investigated. Circulating troponin levels were quantified across groups (Fig. 6). Hb-exposed males exhibited significantly higher troponin levels than estrous and non-estrous females. Additionally, troponin levels were significantly different between estrous and non-estrous females, indicating that hormonal cycling may influence the extent of cardiac injury following Hb exposure. Females exhibited lower levels of circulating troponin when compared to their controls, while males showed a significant increase compared to their controls.

**Fig. 7.**
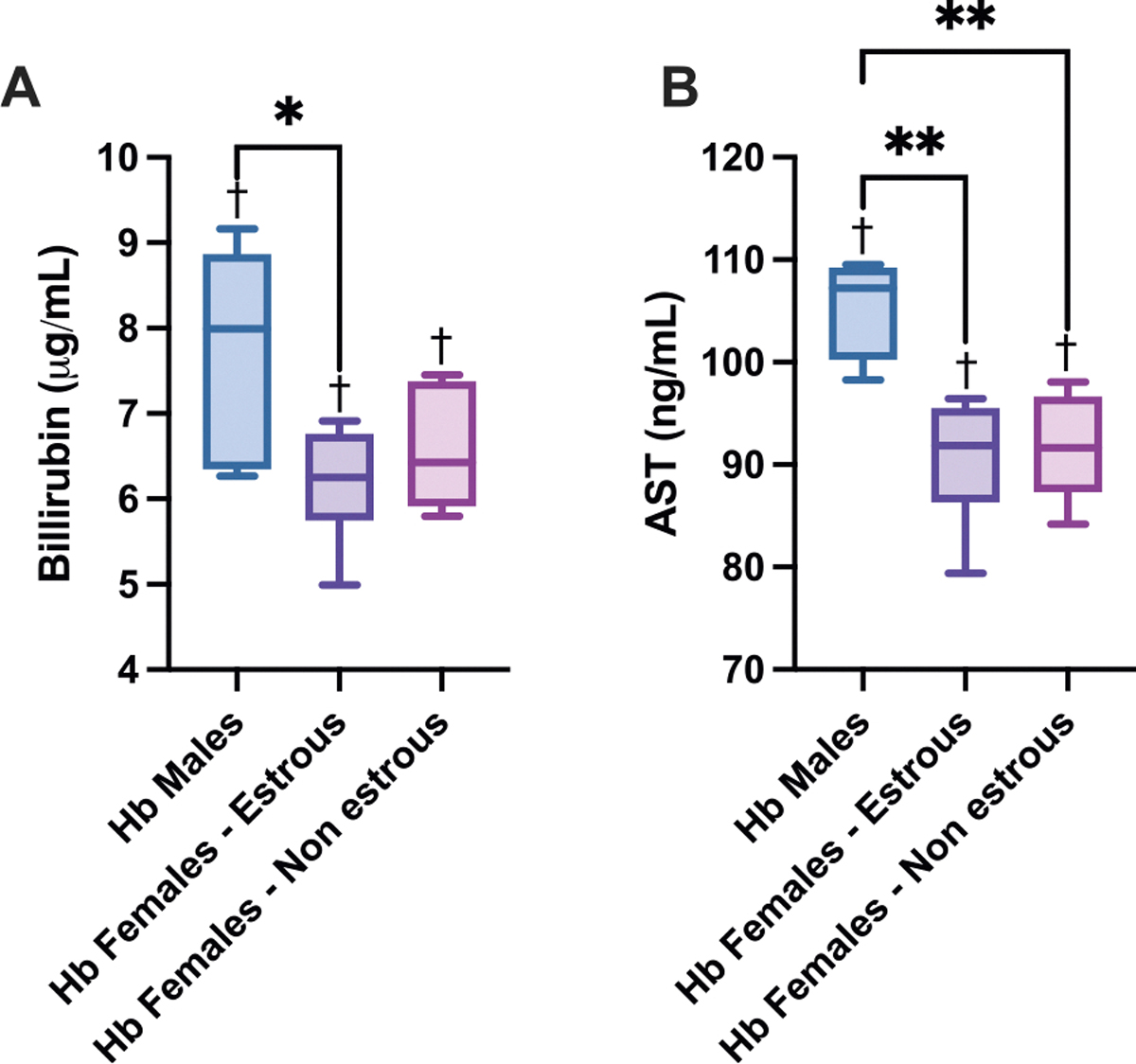
Liver Functional Measurements. **A)** Circulating bilirubin and **B)** AST levels 24 h after Hb infusion in male, estrous female, and non-estrous female rats. Males showed significantly higher bilirubin and AST levels compared to estrous females, while non-estrous females exhibited intermediate levels. Hb exposure significantly elevated both markers compared to their respective control groups (baseline). Statistical significance (p < 0.05) between experimental groups is marked by * and compared to the control group is indicated by †.

Moreover, to understand liver toxicity, injury markers were measured following Hb exposure, with sex and estrous cycle status influencing the degree of damage (Fig. 7). Circulating bilirubin levels were highest in males and significantly elevated compared to both estrous and non-estrous females. Estrous females had the lowest bilirubin concentrations, indicating improved hepatic clearance or reduced burden of hemolytic byproducts. Similarly, aspartate aminotransferase (AST), a marker of hepatocellular injury, was significantly higher in males relative to both female groups. AST levels were lowest in estrous females, further supporting the protective role of hormonal cycling. Across all Hb-exposed groups, bilirubin and AST levels were significantly increased compared to their respective controls, suggesting that cell-free Hb induces hepatic stress regardless of sex. Additional markers, including plasma inflammatory markers and heme/iron quantification due to Hb exposure, can be found in the supplemental materials (see Supplementary Figures S4 and S5).

It is important to note that baseline values were consistent across all control groups, with no significant differences observed. This indicates that the differences seen among the Hb-treated groups are not due to initial disparities but rather reflect sex-specific physiological responses to Hb.

## 4. Discussion

This study examined the effects of acute Hb-induced toxicity on renal function, specifically assessing GFR and molecular markers of kidney injury in male, estrous-phase female, and non-estrous-phase female subjects. Our results indicate that while all groups experienced significant injury in the acute setting, females in the estrous phase exhibited the least pronounced decline in GFR and the lowest levels of molecular markers associated with injury and inflammation. These findings suggest a potential protective effect linked to hormonal status. While some variability in estrogen levels was noted among estrous females, the overall trend of higher estrogen levels in this group aligns with the expected hormonal profile of the estrous phase. This supports existing literature highlighting estrogen’s anti-inflammatory and cytoprotective properties in systemic injury models [29,31,34].

Furthermore, our results demonstrate that the observed changes in GFR are primarily attributable to Hb toxicity. No significant differences were found in hemodynamic parameters such as MAP and HR among the groups. This eliminates systemic variables as external factors affecting GFR, strengthening the conclusion that biological sex and hormonal status influence the animal’s susceptibility to Hb-induced renal dysfunction. Moreover, the functional preservation in estrous-phase females was supported by a variety of injury biomarkers. KIM-1 levels were significantly lower in this group, indicating reduced proximal tubular damage. Similarly, urinary creatinine and NGAL levels were reduced, suggesting preserved tubular function and attenuated oxidative stress. Inflammatory cytokines, IL-1β and IL-18, both downstream products of the NLRP3 inflammasome, were also significantly lower in estrous females compared to males and non-estrous females, reinforcing the hypothesis that estrogen suppresses inflammation in Hb toxicity.

The current literature highlights estrogen’s nephroprotective and cardioprotective properties [29,32,33,42,43]. However, its specific role in mitigating Hb toxicity remained unclear. Clinical observation has reported that male patients with hemolytic anemias experience poorer outcomes during hospitalization compared to females, which may reflect a protective advantage in females, potentially influenced by hormonal differences [44]. However, given the complexity of hemolytic anemias and their various complications, the precise mechanisms of Hb toxicity defense have not been fully elucidated. Previous studies have shown that estrogen can inhibit the NLRP3 inflammasome pathway, preventing downstream apoptotic signaling, reducing ROS activation to limit inflammation, and enhancing nitric oxide (NO) production to prevent excessive vasoconstriction [32,45,46]. Estrogen mediates protective effects primarily through binding to its nuclear receptors, estrogen receptor alpha (ERα) and beta (ERβ), which function as ligand-activated transcription factors [46,47]. Upon activation, these receptors translocate to the nucleus, where they suppress the transcription of pro-inflammatory genes, including those regulated by NF-κB, the priming step for NLRP3 activation [47]. Additionally, estrogen modulates mitochondrial function by reducing mitochondrial-derived ROS, which are necessary for NLRP3 inflammasome assembly [18,22,48]. Estrogen also enhances the expression of antioxidant enzymes by upregulating the Nrf2 pathway, which further limits oxidative stress and inflammasome activation [48–50]. Collectively, these mechanisms converge to limit the activation of the NLRP3 complex, thereby reducing IL-1β/IL-18 production, limiting pyroptosis, and attenuating tissue injury during inflammatory conditions [18,22,23,51].

Our findings align with these protective mechanisms, as females in the estrous cycle maintained GFR comparable to their controls, exhibited lower biomarker levels of inflammasome activation, and had reduced markers of tubular injury and systemic inflammation. In contrast, males and non-estrous females displayed impaired GFR recovery and elevated markers of injury, suggesting that estrogen levels may determine the extent of protection. The similarity in injury patterns between males and non-estrous females may be due to their hormonal environments. During the non-estrous phase, circulating estrogen levels significantly decrease, which reduces their anti-inflammatory and cytoprotective effects. As a result, non-estrous females may lose the protective hormonal benefits present during the estrous phase, making them vulnerable to injury caused by cell-free Hb. This phase-dependent hormonal fluctuation explains why non-estrous females exhibit similar injury profiles to males. Finally, although Hb exposure resulted in multiorgan toxicity across all groups, females in the estrous phase consistently exhibited reduced levels of injury and inflammation in the kidney, liver, plasma, and urine. Lower circulating troponin I and AST levels, along with reduced bilirubin, support a systemic protective effect mediated by hormonal status.

Epidemiological and clinical studies have consistently shown that biological sex influences susceptibility to and recovery from acute kidney injury (AKI) [26,30,52]. Women, particularly of reproductive age, often exhibit less severe AKI and show a more favorable recovery compared to men, potentially due to estrogen’s protective modulation of oxidative and inflammatory [26,30,52]. Our observation that estrous-phase female rats show preserved GFR and reduced expression of injury markers after Hb exposure reflects these clinical trends. Rats experience a 4–5 day estrous cycle, while humans experience a menstrual cycle of approximately 28 days; however, both have cyclic hormonal fluctuations. In humans, circulating estrogen levels peak during the late follicular and mid-luteal phases. These high-estrogen phases may confer temporary renal protection, similar to the estrous phase in our animal model. This parallel supports the idea that estrogen-mediated protection is not exclusive to rodents and may be relevant to women’s menstrual cycle phases in clinical settings.

In the clinical hematology context, these findings are relevant for a multitude of reasons. Hemolytic anemias are one of the acquired forms of chronic hemolysis resulting from genetic modifications, leading to an increased amount of RBC lysis [3,53]. Understanding the implications of Hb damage could improve targeted therapeutics for hemolytic anemia patients who are more susceptible to immediate danger. Our findings suggest that individuals with lower estrogen production are more vulnerable to Hb-induced renal injury, which in the general population would include prepubertal females, post-menopausal females, and males. Moreover, understanding the inherent toxicity response of Hb and how hormonal status influences the response to Hb toxicity becomes relevant in transfusion medicine. Aging RBC units accumulate cell-free Hb over time due to ongoing hemolysis [54,55]. For at-risk populations, transfusing older blood may increase exposure to toxic levels of Hb. The known impacts of acute Hb toxicity can help create clearer guidelines in the medical field to prevent susceptible groups from receiving aged blood and developing Hb toxicity in acute settings.

A key limitation of this study is the introduction of Hb into healthy animals. In more clinically relevant scenarios, patients experiencing Hb toxicity often have underlying comorbidities that can exacerbate renal injury and worsen overall outcomes [44,53,56]. Additionally, in physiological settings, cell-free Hb is typically released alongside RBC fragments, which can exacerbate tissue damage and inflammation, amplifying the effects observed in this study [57–59]. Moreover, our study is limited by the temporal resolution of GFR measurements, as measurements were performed at baseline, 2 and 24 h post Hb exposure. While additional intermediate or later timepoints could provide a more detailed temporal profile of injury and recovery, it is challenging to continuously monitor GFR, and our primary objective was to compare susceptibility to Hb-induced renal dysfunction across sex and hormonal groups during different physiologically relevant phases. An additional limitation is the use of rodents as the animal model. Unlike humans, rodents endogenously produce ascorbic acid, which serves as an antioxidant, thus reducing Hb oxidation, and may provide additional protection against oxidative stress [60,61]. This intrinsic defense mechanism could result in lower levels of injury compared to those expected in human patients, highlighting a potential species-specific limitation of our findings. Furthermore, while our data suggest that estrogen offers protection against Hb-induced renal injury through suppression of inflammatory pathways such as the NLRP3 inflammasome, this conclusion remains correlative. Future studies will need to incorporate pharmacological inhibitors of NLRP3 and estrogen receptor modulation to test the direct role of estrogen in inflammasome signaling.

In conclusion, our study highlights that biological sex plays a significant role in potentially modulating patient outcomes following acute Hb exposure. This highlights the importance of testing therapeutics and interventions across diverse patient populations and adjusting them based on susceptibility to injury, co-morbidities associated with biological sex, and even hormonal fluctuations across different life stages. Future studies should focus on the effects of chronic Hb exposure to better mimic other real-life scenarios, such as those seen in hemolytic anemias, and further explore the protective role of estrogen and sex-based susceptibility. Additionally, models incorporating permanent hormonal status, such as ovariectomized, sustained high and low estrogen levels, will be valuable for distinguishing transient hormonal fluctuations from long-term hormonal influences on injury outcomes.

## Supplementary Material

supplemental

## Data Availability

Data will be made available on request.

## Appendix A. Supporting information

Supplementary data associated with this article can be found in the online version at doi:10.1016/j.biopha.2025.118461.
