# Supplementary material for: Sex-specific glomerular filtration rate changes in response to acute hemoglobin exposure
Source: Biomed Pharmacother. Author manuscript; Available in PMC 2026 Feb 19. (PMC12919708; doi:10.1016/j.biopha.2025.118461)
Supplement: supplemental [file NIHMS2132013-supplement-supplemental.pptx]

## Slide 1
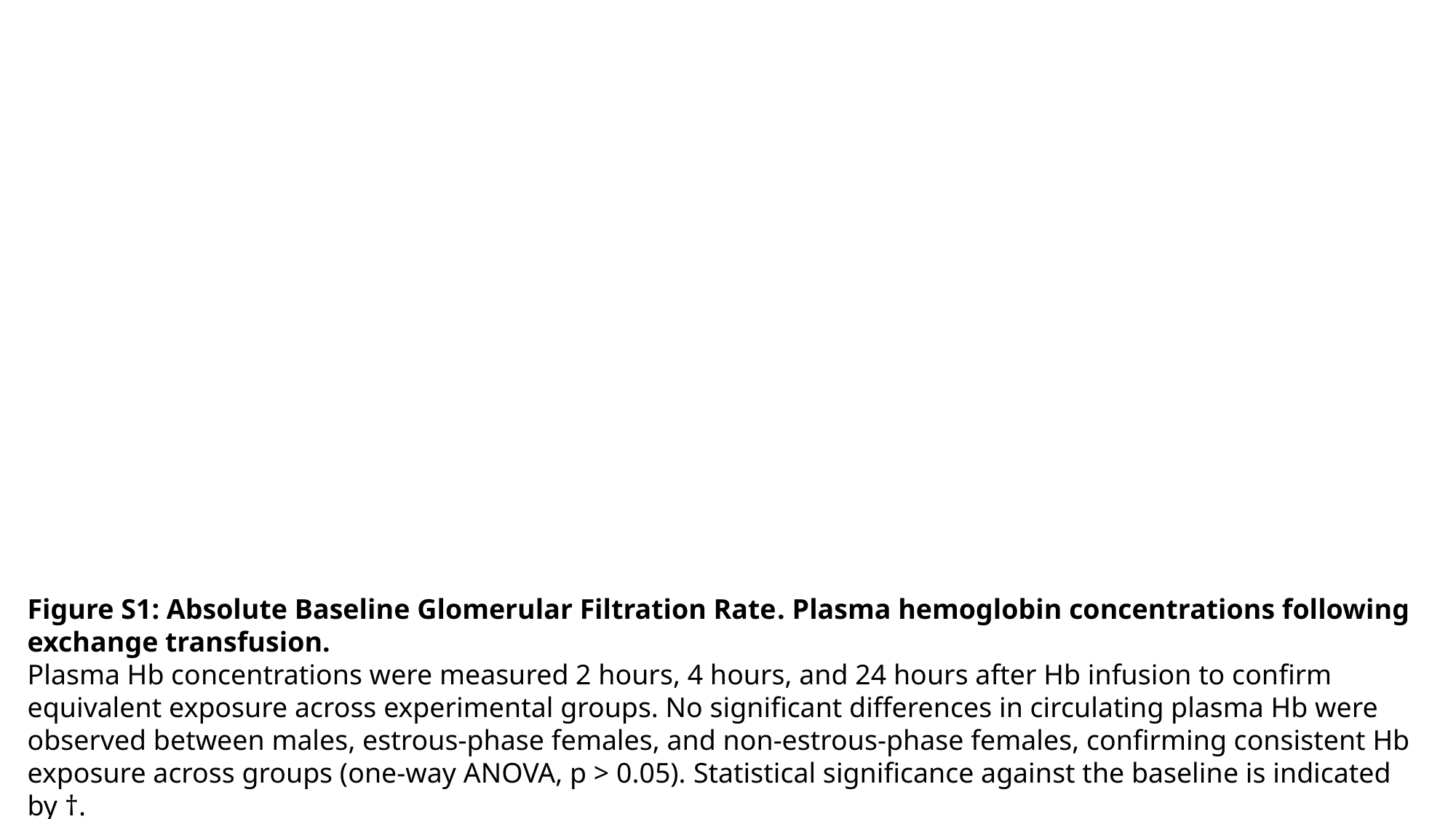

Figure S1: Absolute Baseline Glomerular Filtration Rate. Plasma hemoglobin concentrations following exchange transfusion.
Plasma Hb concentrations were measured 2 hours, 4 hours, and 24 hours after Hb infusion to confirm equivalent exposure across experimental groups. No significant differences in circulating plasma Hb were observed between males, estrous-phase females, and non-estrous-phase females, confirming consistent Hb exposure across groups (one-way ANOVA, p > 0.05). Statistical significance against the baseline is indicated by †.

## Slide 2
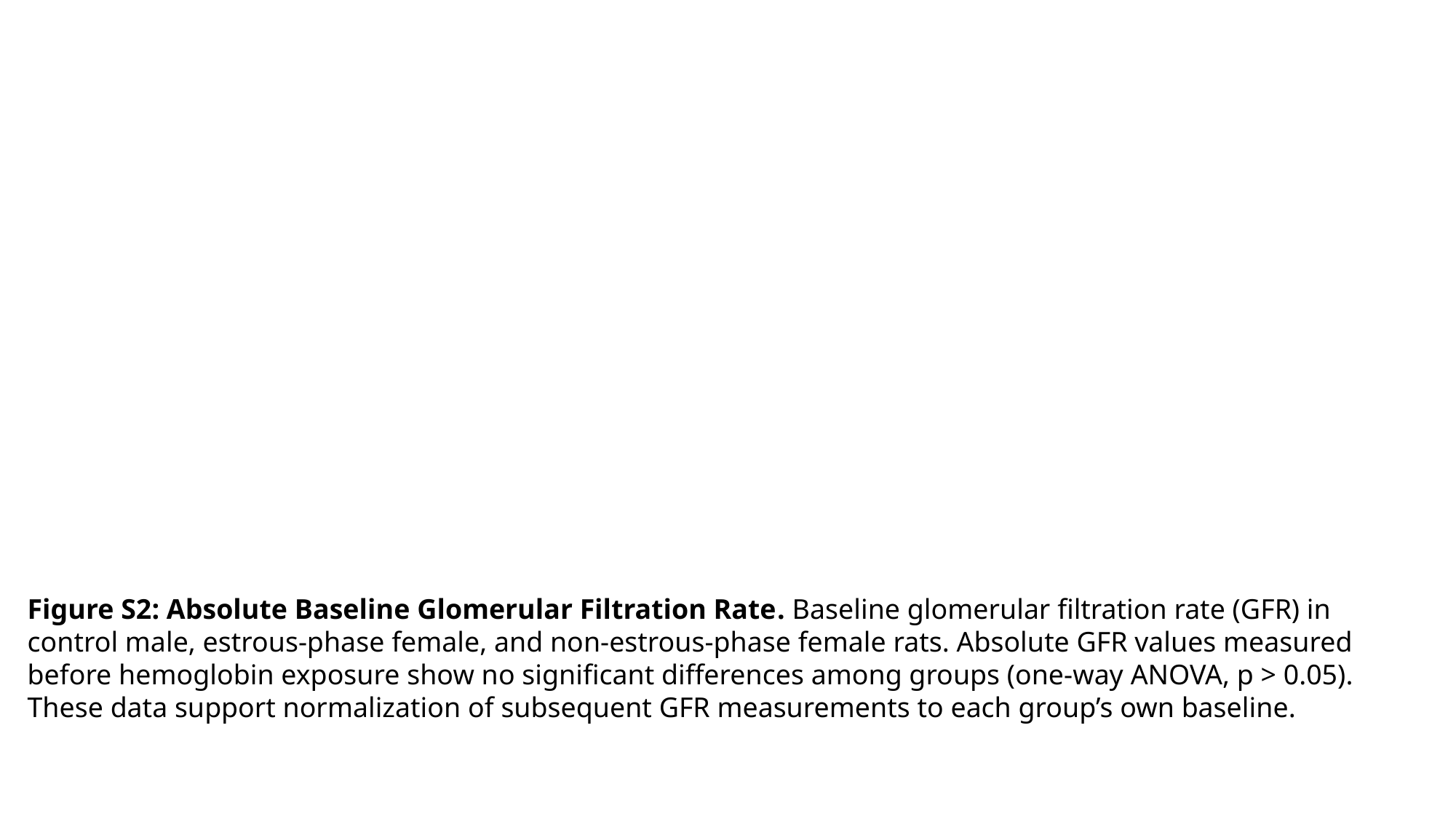

Figure S2: Absolute Baseline Glomerular Filtration Rate. Baseline glomerular filtration rate (GFR) in control male, estrous-phase female, and non-estrous-phase female rats. Absolute GFR values measured before hemoglobin exposure show no significant differences among groups (one-way ANOVA, p > 0.05). These data support normalization of subsequent GFR measurements to each group’s own baseline.

## Slide 3
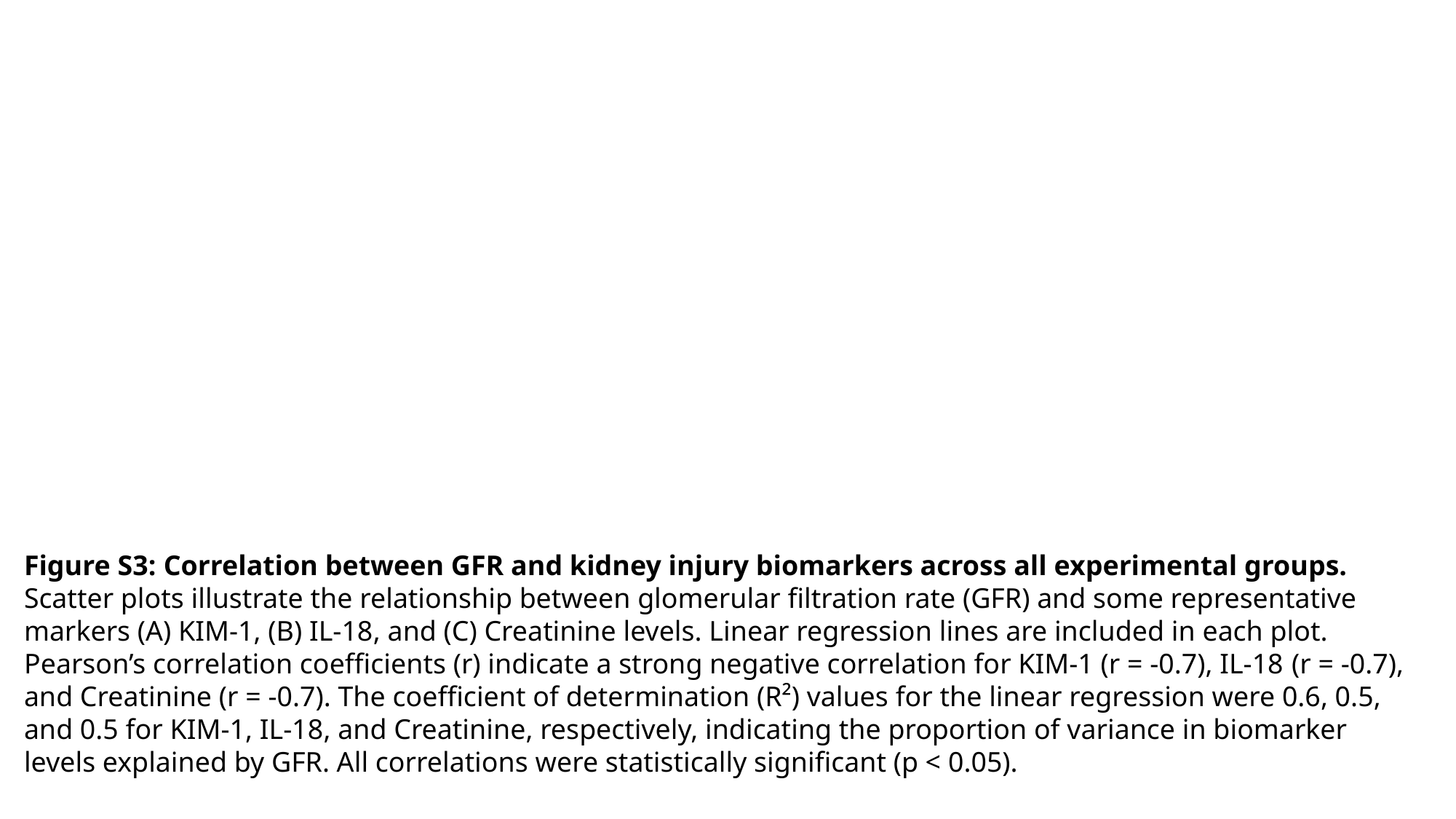

Figure S3: Correlation between GFR and kidney injury biomarkers across all experimental groups.
Scatter plots illustrate the relationship between glomerular filtration rate (GFR) and some representative markers (A) KIM-1, (B) IL-18, and (C) Creatinine levels. Linear regression lines are included in each plot. Pearson’s correlation coefficients (r) indicate a strong negative correlation for KIM-1 (r = -0.7), IL-18 (r = -0.7), and Creatinine (r = -0.7). The coefficient of determination (R²) values for the linear regression were 0.6, 0.5, and 0.5 for KIM-1, IL-18, and Creatinine, respectively, indicating the proportion of variance in biomarker levels explained by GFR. All correlations were statistically significant (p < 0.05).

## Slide 4
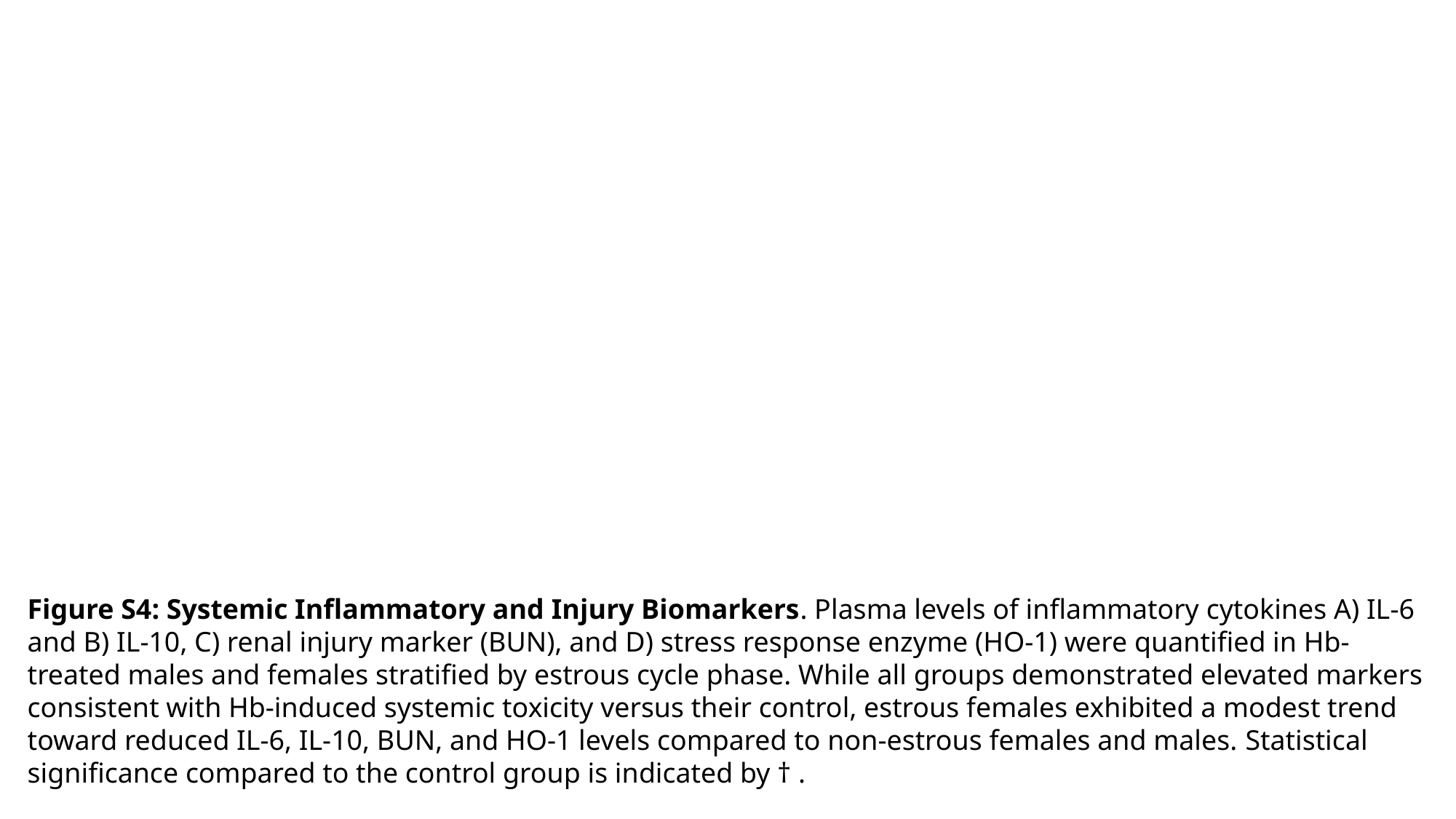

Figure S4: Systemic Inflammatory and Injury Biomarkers. Plasma levels of inflammatory cytokines A) IL-6 and B) IL-10, C) renal injury marker (BUN), and D) stress response enzyme (HO-1) were quantified in Hb-treated males and females stratified by estrous cycle phase. While all groups demonstrated elevated markers consistent with Hb-induced systemic toxicity versus their control, estrous females exhibited a modest trend toward reduced IL-6, IL-10, BUN, and HO-1 levels compared to non-estrous females and males. Statistical significance compared to the control group is indicated by † .

## Slide 5
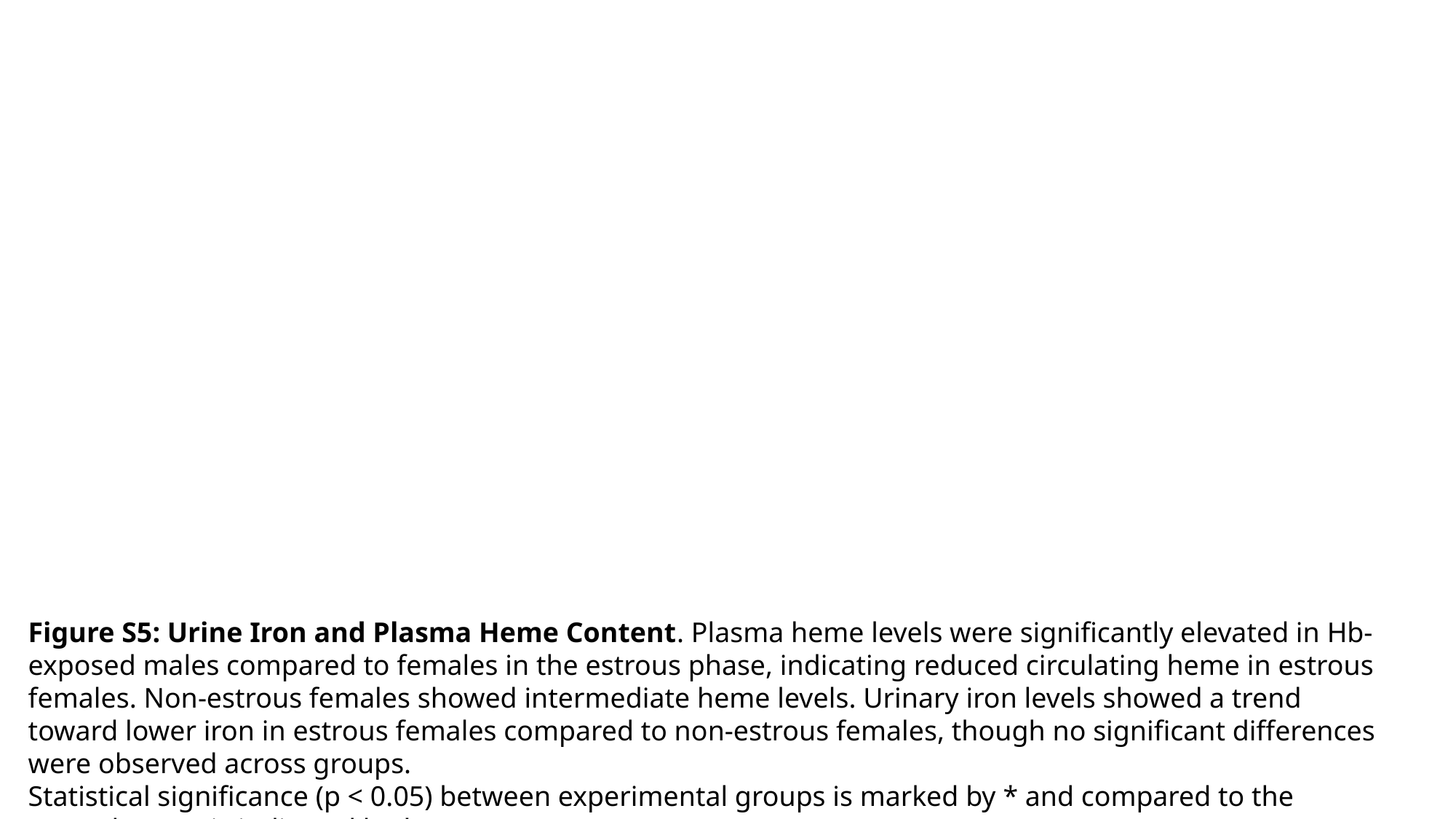

Figure S5: Urine Iron and Plasma Heme Content. Plasma heme levels were significantly elevated in Hb-exposed males compared to females in the estrous phase, indicating reduced circulating heme in estrous females. Non-estrous females showed intermediate heme levels. Urinary iron levels showed a trend toward lower iron in estrous females compared to non-estrous females, though no significant differences were observed across groups.
Statistical significance (p < 0.05) between experimental groups is marked by * and compared to the control group is indicated by † .
